# Abdominal aortic aneurysm model in swine with bovine pericardium patch

**DOI:** 10.1590/1677-5449.210080

**Published:** 2021-09-01

**Authors:** Sílvio César Perini, Leonardo Henrique Bertolucci, Ana Paula Donadello Martins, Luís Henrique Gil França, Celso Curcio Aveline, Adamastor Humberto Pereira

**Affiliations:** 1 Pontifícia Universidade Católica do Rio Grande do Sul – PUCRS, Porto Alegre, RS, Brasil.; 2 Hospital São Lucas da PUCRS – HSL-PUCRS, Porto Alegre, RS, Brasil.; 3 Universidade Federal do Rio Grande do Sul – UFRGS, Porto Alegre, RS, Brasil.; 4 Hospital de Clínicas de Porto Alegre – HCPA, Porto Alegre, RS, Brasil.

**Keywords:** abdominal aortic aneurysm, aortic aneurysm, aneurysm, aneurisma de aorta abdominal, aneurisma de aorta, aneurisma

## Abstract

**Background:**

Aneurysm repair using endovascular techniques has grown in importance as materials have improved. Studies of endovascular prostheses require experimental models that reproduce anatomic and pathophysiological characteristics of human aneurysms.

**Objectives:**

To describe a porcine model of abdominal aortic aneurysm.

**Methods:**

This prospective cohort study used eleven Large White female pigs with a mean age of 12 weeks in two study phases. In phase I, the aneurysm was produced with a bovine pericardium patch by retroperitoneal surgery conducted under general anesthesia. In phase II, 15 days later, the animals underwent arteriography and were then euthanized before specimens were removed for histological analysis.

**Results:**

Formation of parietal thrombus was observed in all animals. Microscopic analysis showed calcifications around thrombus in 82% of the animals. There was lymphoplasmacytic infiltration in the graft and adjacent area, with fibrosis in nine animals. Three pigs had substantial myointimal thickening, and eight had microcalcifications. Mortality was zero, and there were no ruptures, ischemia, or surgery site infections.

**Conclusions:**

This is a unique model, using inexpensive, biocompatible material. Bovine pericardium is easy for the surgeon to handle and has very similar characteristics to autologous tissue in terms of integration with the cell wall.

## Resumo

**Contexto:**

A correção de um aneurisma por meio de técnicas endovasculares tem ganhado importância com o aprimoramento dos materiais. O estudo das próteses endovasculares requer modelos experimentais que reproduzam as características anatômicas e fisiopatológicas dos aneurismas humanos.

**Objetivos:**

Descrever um modelo suíno de aneurisma de aorta abdominal.

**Métodos:**

Este estudo de coorte prospectivo utilizou 11 porcas da raça *Large White* com idade média de 12 semanas nas duas fases do estudo. Na fase I, o aneurisma foi produzido com um saco de pericárdio bovino através de cirurgia por via retroperitoneal sob anestesia geral; na fase II, 15 dias após a fase I, os animais foram submetidos a arteriografia e, posteriormente, a eutanásia, sendo os espécimes retirados para análise histológica.

**Resultados:**

A formação de trombo parietal foi observada em todos os animais. A análise microscópica mostrou calcificações ao redor do trombo em 82% dos animais. Havia infiltrado linfoplasmocitário no enxerto e na área adjacente, com fibrose em nove animais. Três porcos tinham espessamento miointimal substancial, e oito tinham microcalcificações. A mortalidade foi zero, e não houve rupturas, isquemia ou infecção no local da cirurgia.

**Conclusões:**

É um modelo único, com material biocompatível e de baixo custo. O pericárdio bovino é de fácil manuseio pelo cirurgião e possui características muito semelhantes ao tecido autólogo em sua integração com a parede celular.

## INTRODUCTION

The natural history of abdominal aortic aneurysms (AAA) is to progress to rupture, a condition that is associated with a high mortality rate. AAA patients often have comorbidities that increase the risks inherent in surgical treatment, which limits its indications in many cases.^1^ Aneurysms can be treated by open surgery or endovascular repair. The endovascular technique consists of placing an endograft through a minimally invasive operation, thus reducing the morbidity and mortality associated with the procedure.^1^

Both techniques alter the natural history of the disease, limiting expansion by excluding the diseased aortic segment from the circulatory system and, therefore, preventing rupture in 90 to 98% of cases.^1^^,^^2^ However, after endovascular repair, complications related to the devices employed can occur, such as leaks (endoleaks), migration, and fracture of the metal mesh.^3^^-^5

Endografts and the associated catheters, guidewires, and delivery systems have improved over the last three decades. Endoprostheses have undergone changes in shape, size, and lining material, in search of the ideal device. However, studies with these devices require experimental models that must present anatomical and pathophysiological characteristics that emulate aneurysms in humans^6^^-^8 and the animals most often used for these models are dogs and pigs.^9^^-^13

There are animal models that use patches of different materials such as veins, muscular fascia, or synthetic materials such as Dacron, Polytetrafluoroethylene (PTFE), and other models are based on destruction of lamellae of the tunic media.^7^^,^^11^^-^16 However, most of these experimental models are expensive and are associated with high rates of perioperative morbidity and mortality.^6^^,^^13^

The purpose of this study was to evaluate the histopathological characteristics, lesion stability in terms of rupture, reproducibility, morbidity, mortality, and patency of the aorta and its collateral branches in a new aneurysm model using bovine pericardium.

## METHODS

This is a prospective cohort study using eleven female Large White pigs aged 12 weeks and weighing between 20 and 25 kg. This experimental study was approved by the Ethics and Bioethics Committee at the Hospital de Clínicas de Porto Alegre Medical School, Universidade Federal do Rio Grande do Sul, under protocol 04.095. Before starting the project, a pilot study was carried out with two pigs to evaluate the anesthetic technique and the proposed surgical approach. The study was conducted in the Centro de Cirurgia Experimental at the Hospital de Clínicas de Porto Alegre.

The study was divided into two distinct parts: in phase I, an abdominal aortic aneurysm was made with a bovine pericardium patch. In phase II, 15 days after phase I, the animals were studied by aortography and then they were euthanized and material was removed for the anatomopathological study.

**Figure 1:**
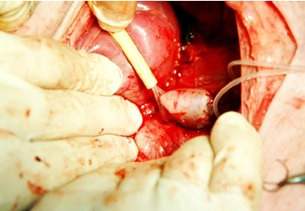
Saccular aneurysm after blood flow is released.

Phase I: in preparation for the anesthetic technique, the animals were weighed the night before the surgery and started on a 10-hour fast. They received pre-anesthetic induction with ketamine at a dosage of 10mg/kg and xylazine 2%, at a dosage of 0.5 to 1mg/kg, intramuscularly. During surgery, continuous infusion of saline solution was maintained by catheterization of the dorsal vein of the ear. The pulse oximeter was placed in the contralateral ear. The animals were given antibiotic prophylaxis with 1g of Cefazolin, the skin was degermed and the sterile field was prepared. Anesthesia was maintained by inhalation with 2% Isoflurane and oxygen at a rate of 0.5 L/min, continuously, through a mask with a closed system for rebreathing. Locoregional anesthesia was obtained by infiltration of the superficial and deep layers of the incision line with 10 ml of 0.5% bupivacaine. Regarding the surgical technique, the aorta was approached via a left retroperitoneal access through an angled incision about 3 cm below the costal margin to the outer margin of the left abdominal rectus sheath. The aorta was exposed in the segment between the renal arteries and its terminal branches, keeping the posterolateral lumbar branches intact and controlled with false ligatures. Anticoagulation was performed with heparin 100U/kg of body weight, before clamping the aorta and performing arteriotomy in the anterior wall, 3.0 cm longitudinally, below the renal arteries. A glutaraldehyde-treated bovine pericardium patch was washed with saline solution, and cut to 3.0X6.0 cm. It was then sutured to the aortotomy with a continuous 6-0 polypropylene suture. After removal of clamps from the aorta and its collaterals, any bleeding failures observed along the suture line were corrected with additional stitches. A successful procedure was confirmed by visualization and palpation of the aneurysm, the proximal aorta, its terminal portion, collateral branches, and femoral arteries (Figure 1). The animals were monitored for signs of pain, discomfort, dyspnea, limb weakness, bleeding, and pale membranes. Analgesia was performed as needed, with dipyrone, intramuscularly by a veterinarian at the animal research center. After recovery, the animals were placed in stalls and fed with an appropriate feed for their species and weight (VIPAL®, Brazil).

In phase II of the experiment, the same anesthetic regimen was used as in phase I. The superficial femoral artery was approached via a transverse incision in the left inguinal region, just below the inguinal ligament. Arteriography was performed using a 6-Fr introducer system and 5-Fr Pigtail angiographic catheter placed above the emergence of the renal arteries. A 20 mL volume of iodinated contrast was injected and images were acquired with a mobile C arm (General Electric model). The images were printed. The renal and mesenteric arteries, collateral arteries, terminal branches, and the aneurysm made with the bovine pericardium patch were visualized. After arteriography, a lethal dose of potassium chloride was administered for euthanasia. Through a xiphopubic incision, the aneurysmal abdominal aorta was removed en bloc and placed in formaldehyde. Slides were prepared by the researchers, stained with Hematoxylin and Eosin (HE), and analyzed by a single pathologist, with an optical microscope, at the Department of Pathology, Hospital de Clínicas de Porto Alegre, Universidade Federal do Rio Grande do Sul. Sections were cut transversely in the suture planes and the bovine pericardium patch.

In the histological study, parietal inflammatory reaction, thrombus, presence of calcification, and presence of fibromuscular dysplasia of the suture line were evaluated. All slides were analyzed by the same pathologist.

## RESULTS

**Figure 2:**
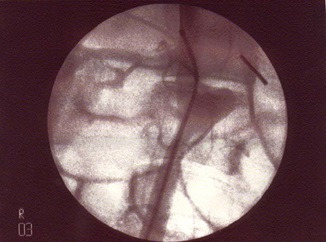
Aortography showing saccular aneurysm and patent lumbar arteries.

**Figure 3:**
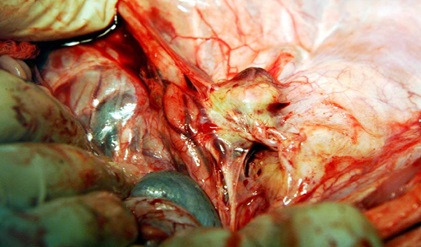
Inflammatory reaction perianeurysm: integration with adjacent structures.

There were no deaths, ruptured aneurysms, or ischemia of limbs or intra-abdominal viscera. There were also no local complications such as dehiscence at the suture of the muscle-aponeurotic wall and skin, hematoma, or infection. On arteriography (Figure 2), fifteen days later, all aneurysms were patent, as were vessels distal of the aneurysm. At the time of removal of the surgical specimen, slight adhesions to adjacent tissues were observed (Figure 3), with no signs of infection in any cases.

**Figure 4:**
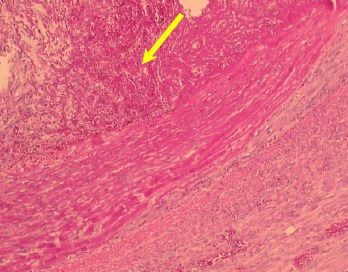
Aneurysm wall thrombosis.

**Figure 5:**
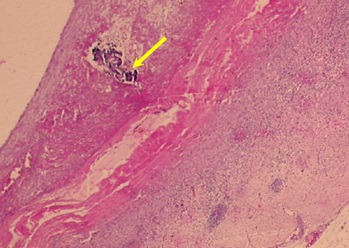
Thrombus with calcification.

Regarding thrombosis, pathological analysis showed that all eleven animals in the study had a thrombus in the aneurysm wall (Figure 4). In seven (63.6%), less than 50% of the vessel lumen was filled, and in the remainder, 50 to 90% of the aneurysmatic aorta diameter was filled. Thrombosis in organization was observed in nine pigs and organized thrombosis was found in two pigs (Table 1). Microcalcifications in the mural thrombus (Figure 5) were seen in 9 animals (81.8%) with 95%CI of 51.73-96.13.

**Table 1:** Presence of trombosis.

**Thrombosis**	**Number (n)**	**Percentage (%)**	**95% Confidence Interval (CI)**
In organization	9	81.8	51.73-96.13
Organized	2	18.2	12.79-66.36
Total	11	100	

**Figure 6:**
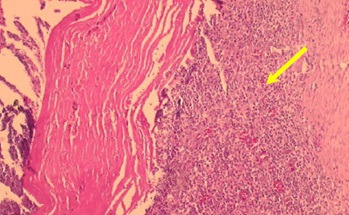
Inflammatory cells and neovascularization.

Regarding inflammation, the acute inflammatory process occurred in all animals (Figure 6), there was chronic inflammation in seven, and fibrosis in nine (Table 2).

**Table 2:** Presence of inflammation or fibrosis.

**Inflammation**	**Number (n)**	**Percentage (%)**	**95%CI**
Acute	11	100.0	76.60-100
Chronic	7	63.6	33.64-87.22
Fibrosis	9	81.8	62.66-99.55

Evident myointimal thickening close to the suture line of the bovine pericardium patch, compared to the portion of the aorta without the lesion, was classified as severe and observed in 3 animals. Eight pigs (72.7%) had microcalcifications in the suture area and in seven (63.6%) there were associated inflammatory cells. No wall necrosis was evident (Table 3).

**Table 3:** Presence of myointimal thickening.

**Myointimal thickening**	**Number (n)**	**Percentage (%)**	**CI 95%**
Severe	3	27.3	7.45-57.81
With microcalcification	8	72.7	42.19-92.55
Associated with inflammation	7	63.6	33.60-87.20
Necrosis	0	-	-

## DISCUSSION

The extant literature refers to the pig as an animal with great similarity to humans in terms of anatomy, fibrinolytic system, coagulation, and response to intimal injury.^17^ In terms of swine breeds, there does not seem to be any superiority or any important differences,^17^^-^19 We therefore opted for female pigs of the Large White breed, because they were readily available and their lineage and kinship are known.

Most studies to develop experimental models that reproduce the anatomical and pathophysiological characteristics of abdominal aortic aneurysms were conducted in the past decade or earlier. The model of aneurysm in dogs described by Economou in 1960,^7^ with resection of the adventitial layer and 70% of the middle layer, is difficult to execute and has very high morbidity and mortality. Dissection of the middle layer seems to be critical to the success of this model: when below 60%, it fails to produce the lesion and when greater than 70%, it tends to rupture. Even the modification of this technique by Mirich,^20^ with the removal of an adventitious layer band followed by balloon dilatation, obtained an unsatisfactory diameter of 46% (36-58%). This model is undoubtedly very difficult to implement and is associated with a high failure rate and high costs.

The model published by Hallisey,^7^ in 1997, also using dogs, was exclusively transluminal. In this model, the aorta was dilated with a Palmaz stent to twice its normal diameter. The lumbar arteries were preserved, maintaining the anatomical condition observed in humans, but the model is expensive and the aneurysm is limited by the metallic mesh of the stent, making it totally different from aneurysms in humans.

Other authors have described models in which the vascular graft replaces a segment of the aorta with an end-to-end suture. The materials most used were Dacron and internal jugular vein treated with glutaraldehyde.^7^^,^^21^ These constructed aneurysms are used to evaluate the performance of introducer systems and release of devices, but cannot be used to study biological responses and integration. In addition, in common with the Elastase Model,^7^^,^^18^ in which an enzyme is used to destroy part of the aortic wall, leaving it weakened and increasing its diameter, the collateral branches of these arteries, such as the lumbar arteries, are not preserved.

Another model frequently used employs patches to enlarge a segment of the aorta, thus forming an aneurysm. Various materials such as muscular fascia, vein, peritoneum, and Dacron have been used.^8^^,^^10^^,^^13^^,^^15^^,^^22^ The greatest advantage of this model is that it preserves the patency of the lumbar arteries.

More recently, creation of saccular aneurysms using Dacron patches has been reported.^23^ This model has the advantage of preserving the lumbar arteries and forming a stable aneurysm due to its low tendency to rupture.

Bovine pericardium is an easy-to-buy and low-cost material, as it is generally removed in meat packing plants and slaughterhouses and preparation with glutaraldehyde is simple.^24^ It is available ready prepared and in various sizes, keeping the cost down.^25^ The disadvantage that has been observed in arterial repairs and in heart valves is calcification.^25^^,^^26^ This was also observed in this study and is actually a positive point, because aneurysms in humans usually exhibit calcification in the wall.^27^

Most of the animal models found used the transperitoneal access to the aorta, which can be associated with high mortality.^13^ The retroperitoneal technique avoids handling of intestinal loops, which is associated with greater morbidity and mortality in pigs.^24^^,^^28^

The cross-sections analyzed included the area of the pericardium patch implant, the suture line and the posterior wall of the aorta. Uflaker^23^ reported thrombus with retraction and calcification in the suture at six weeks and organized thrombus with retraction at the twelfth week. The present study shows an organized thrombus with microcalcifications and calcifications in the suture after two weeks.

In all animals there was formation of mural thrombus and in 82% there were calcifications in the region of the thrombus. Lymphoplasmocytic infiltration was present in the graft and the peri-graft region, with fibrosis in 82% of the animals, and significant myointimal thickening in the suture line in three animals. None of the complications reported in other studies were observed, such as hind leg paralysis,^13^^,^^21^^-^23 death from anesthetic complications,^22^ renal failure,^20^ early rupture,^13^^,^^20^ or thrombosis of the aorta and iliac.^23^

Regarding the anesthesia technique, we chose to use face mask ventilation with room air and oxygen instead of orotracheal intubation. Both methods are widely used in the literature^11^^,^^13^^,^^23^^,^^24^ and the use of a face mask has been shown to be effective, with no risk of glottis edema or need to use atropine to reduce secretions.^24^The bovine pericardium patch has also been described in swine models.^29^ That study used hybrid pigs (crossbred Large White and Landrace), with a transperitoneal approach. Patency of the aorta and the aneurysm patch was confirmed with Doppler ultrasonography and the model of abdominal aortic aneurysm was not analyzed histologically. Formation of a mural thrombus was observed in all animals, with 18% of them showing occlusion of the aneurysm sac, ruling out use in studies for development of new endovascular prostheses. None of the animals in our study presented occlusion of the aneurysm sac.

This study is important because the histopathological analysis showed that the aneurysm created using the bovine pericardium patch exhibits microscopic changes that are similar to those in human abdominal aortic aneurysms. In addition, use of arteriography allowed us to observe the flow in the postoperative period. The limitation of this study is the small number of animals used. In addition, all of the pigs’ aortas had no previous pathological changes.

The bovine pericardium patch model achieves the initial objective of attaining a diameter greater than 50% of the initial aorta and maintaining this vessel and its collateral and terminal branches patent. There are also similar structural findings to aneurysms in humans: parietal and periadventitial inflammatory reaction, thrombus, and calcifications of the wall. The anesthetic and operative techniques developed are satisfactory and there was zero morbidity and mortality in the period evaluated.

In conclusion, the bovine pericardium patch aneurysm is a unique model, which uses inexpensive, biocompatible material and the technology needed for preparation is available in our setting. In addition to its economic advantages, bovine pericardium is easy for the surgeon to handle and has very similar characteristics to autologous tissue in terms of its integration with the cell wall.
